# Neural Androgen Synthesis and Aggression: Insights From a Seasonally Breeding Rodent

**DOI:** 10.3389/fendo.2018.00136

**Published:** 2018-04-04

**Authors:** Kathleen M. Munley, Nikki M. Rendon, Gregory E. Demas

**Affiliations:** Program in Neuroscience, Department of Biology, Center for the Integrative Study of Animal Behavior, Indiana University, Bloomington, IN, United States

**Keywords:** aggression, androgens, brain, dehydroepiandrosterone, steroid-converting enzymes, hamster, seasonality

## Abstract

Aggression is an essential social behavior that promotes survival and reproductive fitness across animal systems. While research on the neuroendocrine mechanisms underlying this complex behavior has traditionally focused on the classic neuroendocrine model, in which circulating gonadal steroids are transported to the brain and directly mediate neural circuits relevant to aggression, recent studies have suggested that this paradigm is oversimplified. Work on seasonal mammals that exhibit territorial aggression outside of the breeding season, such as Siberian hamsters (*Phodopus sungorus*), has been particularly useful in elucidating alternate mechanisms. These animals display elevated levels of aggression during the non-breeding season, in spite of gonadal regression and reduced levels of circulating androgens. Our laboratory has provided considerable evidence that the adrenal hormone precursor dehydroepiandrosterone (DHEA) is important in maintaining aggression in both male and female Siberian hamsters during the non-breeding season, a mechanism that appears to be evolutionarily-conserved in some seasonal rodent and avian species. This review will discuss research on the neuroendocrine mechanisms of aggression in Siberian hamsters, a species that displays robust neural, physiological, and behavioral changes on a seasonal basis. Furthermore, we will address how these findings support a novel neuroendocrine pathway for territorial aggression in seasonal animals, in which adrenal DHEA likely serves as an essential precursor for neural androgen synthesis during the non-breeding season.

## Introduction

Aggression is a well-studied social behavior that is universally exhibited across vertebrates in various social contexts (1, 2). Among conspecifics, aggression is typically displayed when two or more individuals compete for a critical limited resource, such as mates, territories, or food. In seasonally breeding vertebrates, elevated territorial aggression is most often exhibited during the breeding season, when individuals compete for access to resources that will increase their chances of reproductive success. A substantial body of work on male territorial aggression has shown that elevated levels of circulating androgens, including testosterone (T), are correlated with increased aggressive behaviors during the breeding season [reviewed in the studies by Cunningham et al. (3) and Fuxjager et al. (4)]. For example, studies from seasonally breeding birds [(5, 6); but see the study by Apfelbeck et al. (7)]; lizards (8, 9); and rodent species, including mice [(10); but see the study by Trainor and Marler (11)], gerbils (12, 13), and hamsters (14, 15), have demonstrated that castration decreases intermale aggression in a reproductive context, but that this reduction can be alleviated by exogenous T administration. Moreover, individuals with higher endogenous levels of T oftentimes display greater aggression toward conspecifics and may exhibit dominance over animals with lower circulating T levels (16, 17).

Interestingly, some seasonally breeding species exhibit equivalent or increased levels of territorial aggression outside of the breeding season, despite gonadal regression and reduced circulating levels of androgens [reviewed in the study by Soma et al. (18)]. A particularly remarkable example of a species that displays elevated aggression during the non-breeding season is Siberian hamsters (*Phodopus sungorus*), which exhibit robust changes in morphology, physiology, and behavior on a seasonal basis. Siberian hamsters breed during the summer months and cease breeding and undergo gonadal regression, a reduction in body mass, and changes in thermoregulation during the winter months (19). These natural seasonal adaptations can be elicited in the laboratory by housing animals in light cycles that mimic the photoperiods of the breeding and non-breeding seasons (20, 21). For example, animals exposed to short, winter-like days (i.e., >12.5 h of light/day) in the laboratory, which mimic the photoperiod conditions of the non-breeding season, exhibit gonadal regression, a reduction in body mass, and a change in pelage color from brown to white (22, 23). These characteristic changes in physiology are associated with increased levels of aggression in both male (15, 24) and female hamsters (25, 26). Thus, Siberian hamsters are an excellent model for elucidating how seasonal variations in photoperiod can alter the neuroendocrine mechanisms associated with territorial aggression.

## Seasonal Shifts in Neuroendocrine Mechanisms Underlying Aggression

### Pineal Melatonin Regulates Seasonal Changes in Aggression

Although several biotic and abiotic factors vary on a seasonal basis, photoperiod (day length) is the primary environmental cue that is used by animals to shape seasonal shifts in reproductive physiology and social behavior [reviewed in the studies by Prendergast et al. (27) and Walton et al. (28)]. These differences in physiology and behavior are produced *via* a complex neural circuit, which begins with the perception of environmental light *via* retinal ganglion cells and culminates in the transduction of information about day length into a neuroendocrine signal within the pineal gland. The pineal gland secretes melatonin, an indolamine that plays a prominent role in establishing and maintaining biological rhythms, in response to photoperiodic information. Because melatonin secretion tends to be high at night and low during the day, changes in photoperiod cause associated changes in the pattern and duration of melatonin secretion, which convey information about day length to the central nervous system [reviewed in the studies by Bartness et al. (29) and Goldman (30)].

Previous work has implicated pineal melatonin in the modulation of neuroendocrine mechanisms underlying seasonal aggression *via* direct actions on neural substrates, such as the hypothalamus and pituitary gland, and *via* peripheral actions on the adrenal glands and gonads [reviewed in the studies by Haller et al. (31) and Boringer and Nelson (32)]. In seasonally breeding vertebrates, melatonin has regulatory functions at all levels of the hypothalamic–pituitary–gonadal (HPG) and hypothalamic–pituitary–adrenal (HPA) axes and binds to melatonin-type I receptors (MT_1_) located on the hypothalamus (33, 34), anterior pituitary gland (35, 36), gonads (37, 38), and adrenals (39, 40). Collectively, the presence of MT_1_ at different tiers of the HPA and HPG axes suggests that melatonin alters gonadal and adrenal steroid synthesis on a seasonal basis. More specifically, long days induce a lower duration of melatonin secretion, which upregulates the HPG axis relative to the HPA axis to promote gonadal steroid secretion [reviewed in the study by Tsutsui et al. (41)]. Conversely, short days result in prolonged melatonin secretion, which upregulates the HPA axis relative to the HPG axis and elevates adrenal steroid synthesis (26, 42). The action of melatonin at the level of the adrenals is particularly intriguing, since several species of seasonally breeding animals that display year-round territorial aggression increase serum dehydroepiandrosterone (DHEA), an adrenal androgen, during the non-breeding season.

### DHEA Promotes Territorial Aggression During the Non-Breeding Season

In recent years, it has become increasingly evident that DHEA is an important modulator of seasonal aggression in birds and rodents [reviewed in the study by Soma et al. (43)]. DHEA is an androgen that is secreted by the adrenal cortex in certain mammals, including hamsters and squirrels (15, 44). Although DHEA is synthesized peripherally, circulating DHEA is capable of passing through the blood–brain barrier and can be metabolized to active androgens and estrogens, such as T and estradiol (E_2_), *via* a multi-step conversion in brain regions that express the appropriate steroidogenic enzymes (45, 46). Alternatively, some seasonally breeding animals are capable of steroid synthesis *de novo* from cholesterol, and these “neurosteroids” can bind directly to androgen receptor (AR) and estrogen receptors (ERα and ERβ) to regulate social behaviors (47, 48). Because DHEA has a low affinity for AR and ER, high endogenous levels of DHEA would be required to activate these receptors and induce changes in behavior (49). Therefore, it is likely that region-specific metabolism of circulating DHEA and/or neurally synthesized DHEA into active androgens and estrogens is primarily responsible for modulating the neural circuits relevant to territorial aggression during the non-breeding season, since these steroids bind with high affinity to AR and ER in neurons and glia.

**Figure 1:**
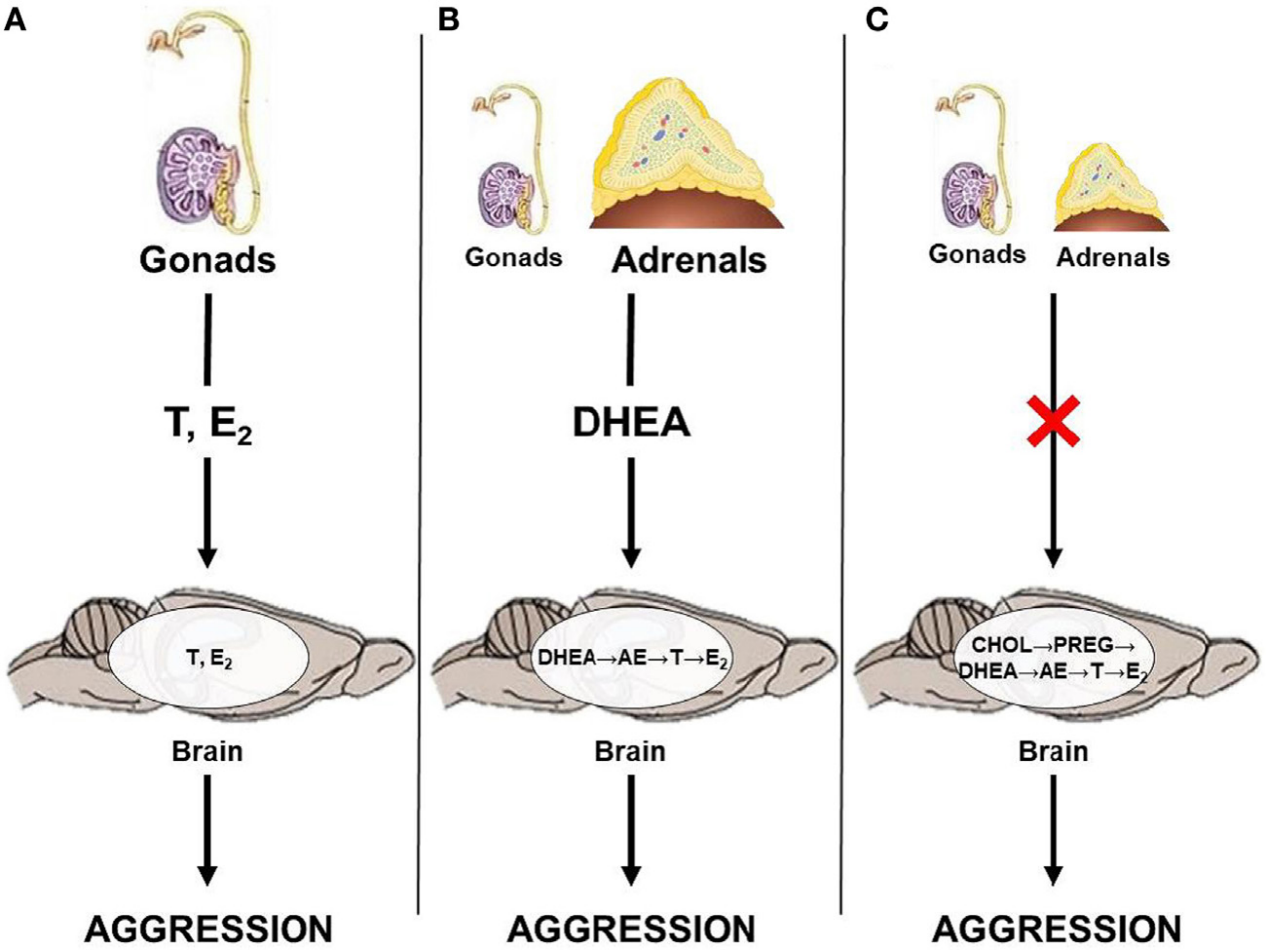
Neuroendocrine pathways by which androgens could affect territorial aggression in seasonally breeding animals. **(A)** Gonadal steroids, such as testosterone (T) and estradiol (E_2_), act directly on the brain; **(B)** adrenal dehydroepiandrosterone (DHEA) is locally converted to T and/or E_2_; and **(C)** steroids are produced *de novo* in the brain from cholesterol (CHOL) *via* conversion to pregnenolone (PREG) and, subsequently, to DHEA, androstenedione (AE), T, and E_2_ in the absence of steroid production from the gonads and adrenal glands.

Results from studies on seasonally breeding animals haveilluminated a few potential neuroendocrine pathways by which androgens can act on relevant neural circuits to modulate seasonal changes in aggression (Figure 1). Briefly, the classical neuroendocrine model proposes that circulating gonadal steroids pass through the blood–brain barrier and act on neural circuits relevant to aggression by directly binding to AR or ER (Figure 1A). This mechanism likely predominates during the breeding season, when animals exhibit elevated levels of circulating androgens. However, outside of the breeding season, circulating gonadal steroids are low and adrenal steroids, such as DHEA, may serve as an important source of neural T and E_2_. Thus, during the non-breeding season, circulating DHEA may serve as a prohormone and be converted to active androgens and estrogens in the brain after passing through the blood–brain barrier (Figure 1B). Alternatively, neurosteroids may be synthesized *de novo* from cholesterol (Figure 1C). These different neuroendocrine mechanisms culminate in region-specific binding of active steroids to AR and ER, respectively, which modulates seasonal changes in territorial aggression.

### Adrenal DHEA as a Source of Neurally-Derived Androgens in Siberian Hamsters

Both male and female Siberian hamsters exhibit pronounced changes in reproductive physiology and territorial aggression across seasonal phenotypes (19). Several studies have demonstrated that male and female hamsters housed in short-day photoperiods display decreased levels of circulating gonadal steroids (23, 25), but increased levels of serum DHEA (15, 26). Furthermore, short-day males and females display elevated levels of aggression (21, 25), and similar increases in aggressive behaviors are observed in long-day animals administered short day-like levels of melatonin (24, 26). Interestingly, short-day males that receive adrenalectomies exhibit reduced levels of aggressive behaviors, yet adrenal demedullation, in which the catecholamine-secreting adrenal medulla is removed, produces no change in aggression (24). Furthermore, photoperiod has no effect on serum or adrenal cortisol content, and cortisol treatment does not affect levels of aggression in short- or long-day males (50). Collectively, these findings suggest that the neuroendocrine control of territorial aggression is dependent on seasonal changes in photoperiod and melatonin secretion, but is independent of gonadal steroids. Moreover, these data suggest that certain adrenocortical steroids may facilitate increases in aggression during the non-breeding season.

Recent studies from our group have focused on elucidating the neuroendocrine mechanisms responsible for increased territorial aggression during the non-breeding season. Specifically, we have examined the potential role of the HPA axis in modulating aggression. We have shown that male and female hamsters housed in short-day photoperiods decrease serum DHEA levels following an aggressive encounter, suggesting that short days may facilitate the conversion of circulating DHEA to T and E_2_ (51, 52). Moreover, short-day female hamsters exhibit changes in adrenal morphology and have significantly higher adrenal DHEA content relative to long-day females. In addition, short- but not long-day females show elevated serum DHEA following adrenocorticotropic hormone (ACTH) challenge, in which the HPA axis is stimulated *via* exogenous administration of ACTH. Exogenous melatonin administration also stimulates adrenal DHEA release and elevates circulating DHEA and aggression in female hamsters (26). Taken together, these findings suggest that melatonin is responsible for coordinating a “seasonal switch” from gonadal to adrenal regulation of aggression by acting directly on the adrenal glands. In addition, these data suggest that DHEA peripherally regulates seasonal aggression.

We have also compared the abundance of gonadal and adrenal steroid receptors in brain regions associated with aggression or reproduction across seasonal phenotypes. We found that photoperiod does not affect glucocorticoid receptor (GR) levels in male hamsters in regions associated with aggressive behavior, including the medial amygdala (MeA) and the medial prefrontal cortex, or in the hippocampus, an area that shows changes in GR levels across photoperiods in other animals (50). In contrast, short-day female hamsters exhibit increases in ERα abundance in brain regions associated with aggression, including the periaqueductal gray (PAG), lateral septum (LS), and bed nucleus of the stria terminalis (BnST), but not in nuclei associated with reproduction, including the preoptic area (POA), arcuate nucleus (ARC), and anteroventral periventricular nucleus of the hypothalamus (53). Likewise, short-day males elevate ERα expression in brain nuclei associated with aggression, including the BnST, MeA, and central amygdala (54). While the presence of steroidogenic enzymes in these brain nuclei has yet to be confirmed, these data suggest that localized variation in neural ERα abundance may be responsible for seasonal changes in territorial aggression.

**Figure 2:**
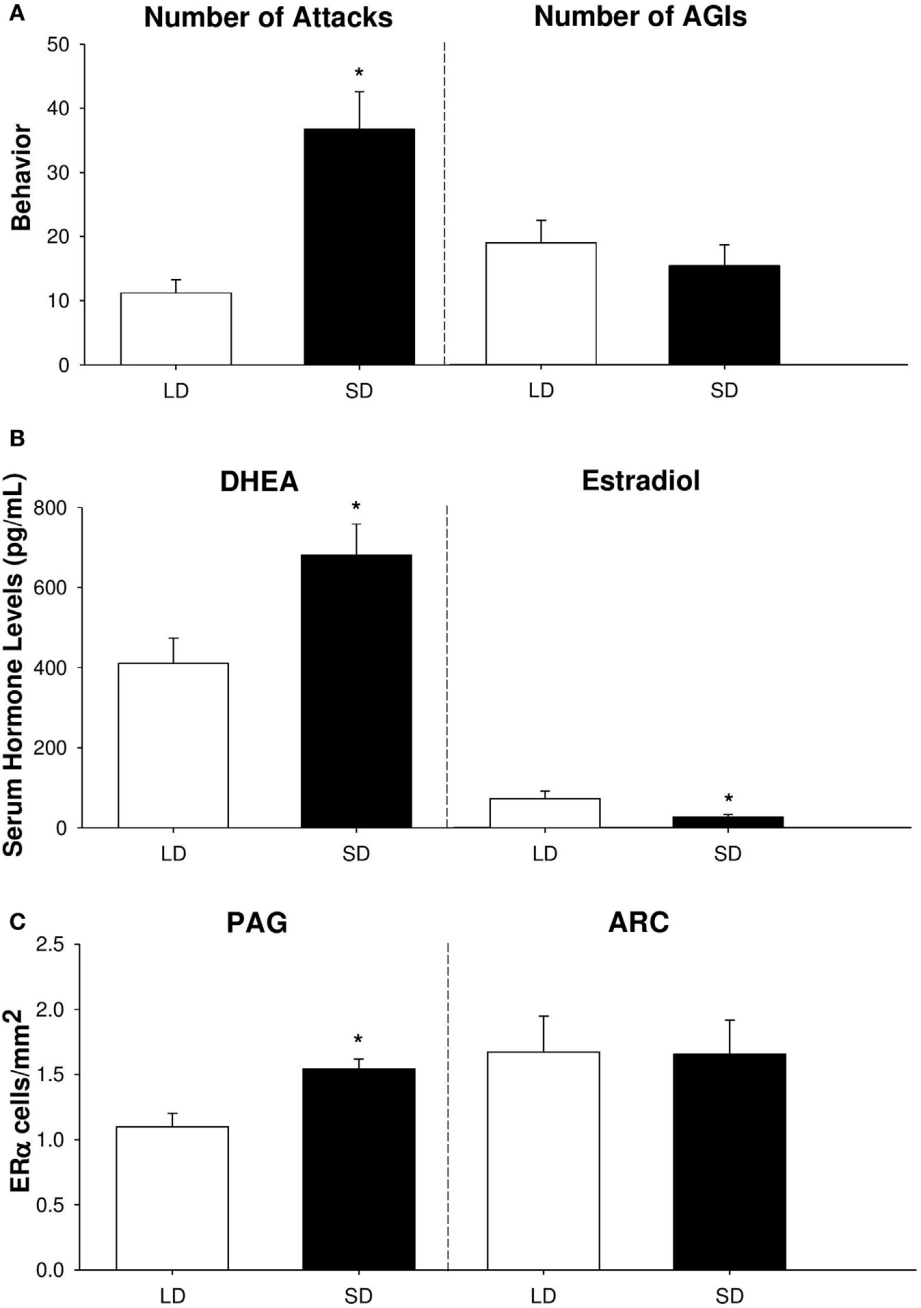
Aggressive behavior, serum hormone profiles, and neural estrogen receptor-α (ERα) abundance differ across reproductive phenotypes of female Siberian hamsters. **(A)** Number of attacks and anogenital investigations (AGIs), **(B)** serum dehydroepiandrosterone (DHEA) and estradiol levels (in pg/mL), and **(C)** ERα cell density (in cells/mm^2^) in the periaqueductal gray (PAG) and arcuate nucleus of the hypothalamus (ARC) in short-day and long-day female hamsters following 10 weeks of photoperiodic treatment. Bar heights represent mean ± SEM. “*” indicates a significant difference between groups (*P* < 0.05). Data are modified and reprinted with permission from the authors (25, 53).

In summary, our findings provide compelling evidence that adrenal DHEA serves as a critical precursor of neurally-derived androgens in non-breeding Siberian hamsters (Figure 2). We have shown that short-day female hamsters display increased levels of aggression, but show no change in other social behaviors, such as investigation [Figure 2A; (53)]. In addition, short-day females increase serum DHEA, yet display reduced circulating levels of E_2_ compared to long-day females [Figure 2B; (25, 53)]. Finally, female hamsters exhibit region-specific changes in ERα abundance, which may be a consequence of increased negative feedback from the HPG axis during the non-breeding season. Specifically, short-day females elevate ERα receptors in regions associated with aggression, such as the PAG, but not in nuclei associated with reproduction, such as the ARC [Figure 2C; (53)]. It is likely that these elevations in ERα abundance upregulate the activity of brain nuclei associated with aggressive behaviors and, consequently, increase territorial aggression during the non-breeding season.

## Neurally-Derived Steroid Synthesis in Other Species

### Mice

Like Siberian hamsters, some species of deer mice (*Peromyscus sp*.) undergo seasonal changes in reproductive physiology and behavior. For example, male beach mice (*P. polionotus*) and deer mice (*P. maniculatus*) increase territorial aggression during the non-breeding season, in spite of gonadal regression and low circulating levels of androgens (55, 56). Intriguingly, California mice (*P. californicus*) do not undergo gonadal regression in response to short-day photoperiods, yet still exhibit elevated levels of aggression during the non-breeding season (57, 58). Together, these data suggest that increases in aggression during the non-breeding season are independent of circulating gonadal steroid levels and that, instead, localized neural androgen and estrogen synthesis may be responsible.

Recent work on the neuroendocrine mechanisms underlying seasonal aggression in deer mice has primarily examined neural ER abundance in brain nuclei associated with aggressive behaviors. Both short-day male beach mice and male deer mice exhibit increases in ERα abundance and expression in the BnST, yet display decreases in ERβ abundance and expression in the BnST and MeA. These changes in ERα- and ERβ-immunoreactivity are correlated with increased levels of aggression (59). Moreover, selective activation of either ERα or ERβ is associated with increased aggression in short-day male beach mice, and these increases in aggression are not correlated with changes in estrogen-dependent gene expression in the BnST and POA (60). While it is unclear whether adrenal DHEA is a source of neural estrogens in this signaling pathway, these findings suggest that ER abundance increases in a region-specific manner and primarily *via* non-genomic pathways to increase territorial aggression during the non-breeding season in *Peromyscus species* that are reproductively responsive to changes in photoperiod.

In contrast, mice that reproduce year-round or are physiologically non-responsive to changes in photoperiod do not exhibit similar changes in steroid hormone synthesis. For example, house mice (*Mus musculus*) decrease aggression in response to exogenous DHEA administration (61, 62). Furthermore, seasonally breeding male California mice, which do not undergo gonadal regression during non-breeding season, still elevate aggression and circulating E_2_ levels in response to short days. However, non-breeding aggression in this species is independent of changes in ERα abundance, as short-day and long-day males show no differences in ERα and ERβ immunostaining in the LS, BnST, MeA, or POA (58, 63). Collectively, these findings indicate that some rodent species may not utilize circulating DHEA to maintain year-round territorial aggression. Additionally, these studies suggest that only seasonally breeding mice that are physiologically responsive to changes in day length alter neural ER abundance to elevate non-breeding aggression.

### Birds

Most avian species have distinct breeding and non-breeding seasons, and many of these species display high levels of territorial aggression throughout the year [reviewed in the study by Soma (64)]. Like seasonally breeding rodents, birds undergo gonadal regression and exhibit pronounced reductions in plasma androgen levels during the non-breeding season, yet still exhibit levels of aggression that are often quantitatively and qualitatively similar to that displayed during the breeding season (65, 66). Moreover, castration of non-breeding male song sparrows has no effect on territorial aggression (67). Thus, while breeding season aggression is mediated by gonadal steroids, territorial aggression exhibited outside of the breeding season may be regulated by non-gonadal steroids in some male songbirds (68, 69).

Several studies have provided evidence that DHEA is an indirect source of neural androgens and estrogens in song sparrows (*Melospiza melodia*) during the non-breeding season. Male song sparrows display elevated circulating DHEA levels during the non-breeding season, which typically match seasonal changes in territorial aggression (66, 70). In addition, non-breeding male song sparrows display elevated plasma DHEA in the jugular vein exiting the brain, but not in the peripherally-located brachial vein, following a simulated territorial intrusion [STI; (71)]. Non-breeding male song sparrows also have higher 3β-hydroxysteroid dehydrogenase (3β-HSD) activity, an enzyme that converts DHEA to the androgen precursor androstenedione, in brain regions associated with aggression, including the LS, BnST, and the taenial amygdala, the avian equivalent of the MeA (72). Furthermore, sparrows exhibit increased aromatase activity, an enzyme that converts T to E_2_, and increased ERα and ERβ mRNA expression in brain nuclei that regulate aggression (73, 74). A more recent study confirmed that neural levels of DHEA, T, and E_2_ are higher in brain tissue than in circulation and that a single STI event alone can induce localized changes in neural DHEA levels (75). Taken together, these data suggest that circulating DHEA is an important source of neurally-derived steroids and that DHEA metabolism in the brain stimulates territorial aggression during the non-breeding season of birds.

Interestingly, there is emerging evidence that this mechanism is evolutionarily-conserved in some tropical bird species, but not others. Non-breeding male spotted antbirds (*Hylophylax n. naevioides*) elevate plasma DHEA, reduce circulating T, and display similar aggressive behaviors compared to breeding males (76, 77). In contrast, non-breeding European nuthatches (*Sitta europaea*) do not elevate aggressive behaviors following exogenous DHEA administration (78), and non-breeding male European starlings (*Sturnus vulgaris*) exhibit lower plasma DHEA levels compared to breeding males (79), indicating that these species maintain year-round territorial aggression independently of DHEA. Intriguingly, inhibiting AR and aromatase activity reduces territorial aggression in breeding, but not non-breeding male European stonechats (*Saxicola torquatus rubicola*), suggesting that steroid hormones only regulate territorial aggression in a breeding context (80, 81). Because the neuroendocrine mechanisms underlying seasonal aggression in songbirds vary considerably across species and context, additional studies in this area of research are warranted.

## Conclusion and Future Directions

Several species of seasonally breeding animals maintain or elevate territorial aggression during the non-breeding season, despite reductions in circulating gonadal steroids. Although our work on Siberian hamsters suggests that non-breeding animals metabolize adrenal DHEA to locally synthesize neural androgens and induce changes in aggressive behaviors, several aspects of these mechanisms have yet to be proven experimentally. Future studies should compare peripheral and central levels of gonadal and adrenal steroids across reproductive phenotypes and investigate whether neurosteroid concentrations and AR and ERβ abundance, in addition to ERα abundance, vary seasonally in brain nuclei associated with aggression. Moreover, little is known about how photoperiod affects the activity of enzymes involved in gonadal steroid synthesis (e.g., 3β-HSD and aromatase), both in the brain and in systemic circulation. It is important that future work addresses this area of research to pinpoint the sex steroid synthesis pathway cascades that are critical in establishing seasonal differences in aggression. Ongoing and future studies in hamsters and other seasonal species will continue to provide important insights into the role of neurally-active steroid hormones in the regulation of aggression.

## Author Contributions

KM and GD drafted the manuscript, with editorial contributions from NR.

## Conflict of Interest Statement

The authors declare no competing interests, financial, or otherwise.
